# Investigating key cell types and molecules dynamics in PyMT mice model of breast cancer through a mathematical model

**DOI:** 10.1371/journal.pcbi.1009953

**Published:** 2022-03-16

**Authors:** Navid Mohammad Mirzaei, Navid Changizi, Alireza Asadpoure, Sumeyye Su, Dilruba Sofia, Zuzana Tatarova, Ioannis K. Zervantonakis, Young Hwan Chang, Leili Shahriyari

**Affiliations:** 1 Department of Mathematics and Statistics, University of Massachusetts Amherst, Amherst, Massachusetts, United States of America; 2 Department of Civil and Environmental Engineering, University of Massachusetts, Dartmouth, Massachusetts, United States of America; 3 Department of Biomedical Engineering and OHSU Center for Spatial Systems Biomedicine (OCSSB), Oregon Health and Science University, Portland, Oregon, United States of America; 4 Department of Bioengineering, UPMC Hillman Cancer Center, University of Pittsburgh, Pittsburgh, Pennsylvania, United States of America; University at Buffalo - The State University of New York, UNITED STATES

## Abstract

The most common kind of cancer among women is breast cancer. Understanding the tumor microenvironment and the interactions between individual cells and cytokines assists us in arriving at more effective treatments. Here, we develop a data-driven mathematical model to investigate the dynamics of key cell types and cytokines involved in breast cancer development. We use time-course gene expression profiles of a mouse model to estimate the relative abundance of cells and cytokines. We then employ a least-squares optimization method to evaluate the model’s parameters based on the mice data. The resulting dynamics of the cells and cytokines obtained from the optimal set of parameters exhibit a decent agreement between the data and predictions. We perform a sensitivity analysis to identify the crucial parameters of the model and then perform a local bifurcation on them. The results reveal a strong connection between adipocytes, IL6, and the cancer population, suggesting them as potential targets for therapies.

## Author summary

One of the outstanding challenges of the mathematical modeling of cancer progression is the existence of many unknown parameters. In this work, we develop a data-driven mathematical model of breast cancer progression by deriving a system of ordinary differential equations for the interaction networks of key cell types and molecules in breast tumors. To overcome the limitations of unknown parameters, we utilize a time course data of a PyMT mice model of breast cancer and estimate parameters using an optimization method. Although the predicted dynamics of cancer and necrotic cells using the obtained values of parameters are in good agreement with the data, the predicted values for a few other variables do not match the data. This might indicate that there are some other key interactions that have not been modeled, and/or there is a noise in the data. The sensitivity and bifurcation analyses show that the most important parameters in controlling the cancer cells population are the proliferation and death rates of cancer cells and adipocytes. These results are in agreement with some biological and clinical studies of breast cancer, which have reported a link between adipocytes and breast cancer progression.

## Introduction

Breast cancer is known to be one of the most common types of cancer in women. In 2021, breast cancer accounted for 50% of all new diagnoses in the USA, with a projected death of 43,600 [1]. Breast cancer can be divided into different subtypes through molecular-level analysis of gene expression patterns. These subtypes are defined as luminal A (LumA), luminal B (LumB), luminal/human epidermal growth factor receptor 2 (HER2), HER2 enriched, basal-like, and triple-negative breast cancer (TNBC) nonbasal [2]. LumA is the most common type with the lowest mortality rate among other subtypes [3]. Most cancer treatments available today focus on killing cancer cells as well as removing them via surgery [4–6]. While these treatments may cure cancer, in some cases, cancer metastasizes to other areas of the body after treatments [7, 8]. Furthermore, it is estimated that 70-80% stage four metastatic breast cancer patients die within five years [9]. Understanding the biology of cancer as a whole intricate system of interactions is crucial for assessing the invasiveness of cancer and obtaining effective treatments.

The tumor microenvironment is a mixture of many cell types and molecules. The importance of the cells and molecules interaction networks within the microenvironment in tumor development has attracted many researchers from many disciplines; whether it is the cancer progression [10–12] or its response to treatments [13–15]. The interactions between immune cells, cancer cells, necrotic cells, and adipocytes result in interesting dynamics and lead to the secretion of important cytokines such as HMGB1, IL12, IL10, and IL6 [16, 17]. These cytokines are often subjects of targeted therapies [18–21].

In-vivo investigation of the tumor microenvironment can be costly and straining for both patients and scientists. Therefore, mouse models have been one of the most popular methods of studying cancers’ initiation and progression. There are many different approaches such as transgenic [22–25], gene targeting [26–28], RNA interference [29–31], and many more to create mouse models. The mammary specific polyomavirus middle T antigen overexpression mouse model (MMTV-PyMT) is the most popular mouse model used in cancer studies, especially in breast cancer which qualifies as a transgenic approach. These models show the signaling of receptor tyrosine kinases commonly activated in many human progressive tumors, including breast cancer [32, 33]. Therefore, they are very suitable as they closely mimic the development of breast cancer in humans.

Mathematical models have enabled scientists to gain insight into biological phenomenon, which are either obscure or costly to experiment. Modeling cardiovascular system [34–36], disease spread [37–39], muscle function [40, 41] and ocular disease [42, 43] are just a few examples of them. Cancer is among one of the most mathematically modeled diseases, some aim to answer questions about cancer as a whole system of biological and chemical interactions [44–47], some investigate the mechanical properties of a cancerous tissue [48, 49], and some others focus on modeling cancer response to different treatments [50–52]. The most desirable features of mathematical models of cancer, including stochastic [53–55] and deterministic models [56–58], are their ability to make good predictions, testing plausible biological hypotheses or generating clinically testable hypothesis. For example, a multiscale model of prostate cancer shows that low androgen levels may increase resistance to hormonal therapy and that treatment with 5*α*-reductase inhibitors may lead to more therapy-resistant cancer cells [59], and a data driven mathematical model predicts the response to FOLFIRI treatment for colon cancer patients [60]. Moreover, an agent-based model [61] of tumor progression indicate that while macrophages existence can increase the size of the tumor, an increase in their infiltration has a reverse effect. In another study, a hybrid agent-based model [62] of ductal carcinoma, which is a common type of breast cancer that starts in cells that line the milk ducts, finds that duct advance rates happen in two phases of an early exponential expansion, followed by a long-term steady linear expansion. Additionally, a free boundary mathematical model of the early detection of recurrences shows a relation between the size of the growing cancer and the total Serum uPAR mass in the cancer [63].

Simple models such as the logistic model and Gompertz model have helped in understanding the growth dynamics of the tumor, predicting the age of the tumor, etc. [64, 65]. These models are dependent on parameters such as proliferation and degradation rates which may depend on cells and molecules interactions in the tumor. Thus, such simple models have the capacity of expanding into more comprehensive models and include many factors through a system of ordinary differential equations (ODEs). This could give a better understanding of how the tumor develops to find optimal treatments [66–70]. However, for mathematical models, obtaining parameter values is always a challenge due to the lack of biological data, especially time course dataset.

In this paper, We use a system of ODEs to describe the dynamics of some key players in the breast tumors microenvironment. We utilize a time course data set collected from three PyMT mice at four different stages of cancer’s progression [71] to estimate the parameters of our proposed mathematical model through an optimization approach. There are several methods for estimating model parameters, including Monte Carlo Hastings and steady-state assumption. Each of these methods has its benefits and drawbacks. Monte Carlo methods, in particular, necessitate a large number of simulations; therefore, they are extremely slow for large-scale problems. Although assuming steady-state evaluates the system parameters straightforwardly, some variables reach the steady-state prematurely and may not correspond with the data in some cases. Here, the least-squares optimization is used to handle parameter estimation in a feasible region for a breast cancer mouse model. Researchers have employed this method to evaluate the system parameters in various disciplines [72–74]. We apply the least-square optimization method on the PyMT mouse time course data set to estimate the parameters of our model. We then assess the sensitivity of our model to its parameters using a sensitivity analysis based on a direct differential method. Finally, we locally investigate the effect of sensitive parameters using bifurcation plots. The results show interesting connections with important biological observations reported in the literature.

## Materials and methods

**Fig 1:**
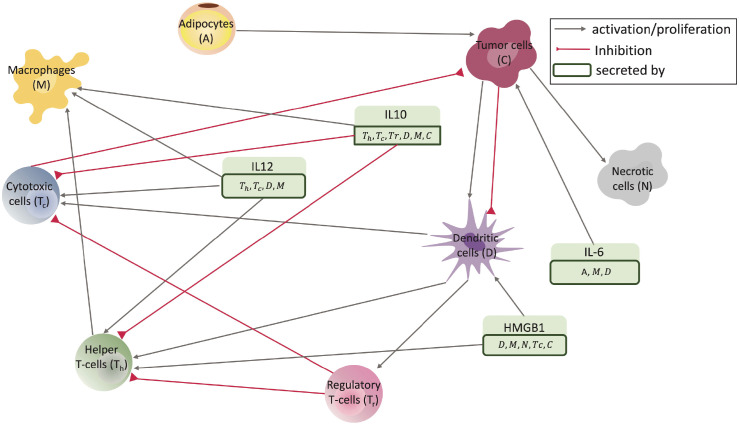
Interaction network. Diagram of interactions between different cell types and molecules in breast tumors for the mouse model, as it has been modeled in this paper. Variables of the model with their descriptions are given in Table 1.

Many cells and cytokines are involved in cancer development, but to avoid complexity, we only consider the most important ones give in Table 1. Fig 1 shows the interaction network between different cell types and molecules used in this paper.

**Table 1:** Mouse data correspondence with variables.

Variable	Name	Data used
*T*_*N*_	Naive T-cells	Combination of CD4 naive and memory resting T-cells and resting NK cells
*T*_*h*_	Helper T-cells	Combination of memory activated CD4 T-cells and follicular helper T-cells
*T*_*C*_	Cytotoxic cells	Combination of CD8 T-cells and activated NK cells
*T*_*r*_	Regulatory T-cells	Regulatory T-cells
*D*_*N*_	Naive dendritic cells	Naive dendritic cells
*D*	Activated dendritic cells	Activated dendritic cells
*M*_*N*_	Naive Macrophages	Combination of Macrophages M0 and Monocytes
*M*	Macrophages	Combination of M1 and M2 Macrophages
*C*	Cancer cells	Estimated
*N*	Necrotic cells	Estimated
*A*	Cancer Associated Adipocytes	Assumed to be twice of the total number of immune cells
*H*	HMGB1	HMGB1 gene expression
*IL*_12_	IL-12	IL12A and IL12B gene expressions
*IL*_10_	IL-10	IL10, IL10RA and IL10RB gene expressions
*IL*_6_	IL-6	IL6, IL6ST and IL6RA gene expressions

Correspondence between the model variables and the gene expression data of the primary tumors and deconvolution results.

### Cells and molecules interaction network—ODE model

#### T-cells

We categorize T-cells into 4 major sub-types: naive (*T*_*N*_), helper (*T*_*h*_), cytotoxic (*T*_*C*_) and regulatory (*T*_*r*_) T-cells; with naive T-cells being the only sub-type, which is not present in the tumor microenvironment and is mainly found in the lymphatic system [75]. Even though, introducing nonlinearity in ODEs for T-cells can prevent issues such as unlimited exponential growth, for simplicity, we just make activation rates of other T-cells proportional to the number of naive T-cells. In this way, we control the system with less complexity. Therefore, we describe the ODEs for helper, cytotoxic and regulatory T-cells prior to that of the naive T-cells.

#### CD4+ helper T-cells (*T*_*h*_)

Dendritic cells [76, 77], HMGB1 [78, 79], and IL-12 [66, 80] activate CD4+ helper T-cells, while regulatory T-cells [81, 82] and IL-10 [83, 84] inhibit them. Therefore, we use the following ODE to describe the dynamic of helper T-cells

$$\begin{array}{c} \begin{array}{cc} \frac{d[T_{h}]}{dt}= & (\lambda_{T_{h}H}\left[H\right]+\lambda_{T_{h}D}\left[D\right]+\lambda_{T_{h}IL_{12}}\left[IL_{12}\right])\left[T_{N}\right]\\ & -(\delta_{T_{h}T_{r}}\left[T_{r}\right]+\delta_{T_{h}IL_{10}}\left[IL_{10}\right]+\delta_{T_{h}})\left[T_{h}\right]. \end{array} \end{array}$$ (1)

#### Cytotoxic T-Cells (*T*_*c*_)

Dendritic cells [85, 86] and IL-12 activate naive CD8+ T-cells [66, 80]. On the other hand, regulatory T-cells [66, 82] and IL-10 [83, 84] suppress Cytotoxic T-Cells functionality. Due to the similarity between natural killer (NK) cells and Cytotoxic T-Cells in directly killing cancer cells, we assume this group includes both CD8+ T-cells and NK cells. Therefore, we model cytotoxic T-cells’ dynamics in the following way.

$$\begin{array}{c} \frac{d[T_{c}]}{dt}=(\lambda_{T_{c}D}\left[D\right]+\lambda_{T_{c}IL_{12}}\left[IL_{12}\right])\left[T_{N}\right]-(\delta_{T_{c}T_{r}}\left[T_{r}\right]+\delta_{T_{c}IL_{10}}\left[IL_{10}\right]+\delta_{T_{c}})\left[T_{c}\right]. \end{array}$$ (2)

#### Regulatory T-Cells (*T*_*r*_)

Dendritic cells stimulate formation [87] and activation of regulatory T-cells [86, 88]. Hence, we have the following equation for the dynamics of T-reg cells.

$$\begin{array}{c} \frac{d[T_{r}]}{dt}=\lambda_{T_{r}D}\left[D\right]\left[T_{N}\right]-\delta_{T_{r}}\left[T_{r}\right]. \end{array}$$ (3)

#### Naive T-Cells (*T*_*N*_)

Combining Eqs (1)–(3) for the activation of naive T-cells and adding an independent naive T-cells production rate $A_{T_{N}}$, we get the following ODE for for naive T-cells

$$\begin{array}{ccc} \frac{d[T_{N}]}{dt} & = & A_{T_{N}}-(\lambda_{T_{h}H}\left[H\right]+\lambda_{T_{h}D}\left[D\right]+\lambda_{T_{h}IL_{12}}\left[IL_{12}\right])\left[T_{N}\right]\\ & - & (\lambda_{T_{c}D}\left[D\right]+\lambda_{T_{c}IL_{12}}\left[IL_{12}\right])\left[T_{N}\right]\\ & - & (\lambda_{T_{r}D}\left[D\right]+\delta_{T_{N}})\left[T_{N}\right]. \end{array}$$ (4)

### Dendritic cells (*D*)

Cancer cells [66, 89] and HMGB1 [67, 90–92] can activate dendritic cells. Moreover, cancer cells may promote natural death of dendritic cells in different ways [86]. Adding $A_{D_{N}}$ as the production rate of naive dendritic cells, we get the following system of equations for naive dendritic cells (*D*_*N*_) and activated dendritic cells (*D*)

$$\begin{array}{ccc} \frac{d[D_{N}]}{dt} & = & A_{D_{N}}-\left(\lambda_{DC}\left[C\right]+\lambda_{DH}\left[H\right]\right)\left[D_{N}\right]-\delta_{D_{N}}\left[D_{N}\right], \end{array}$$ (5)

$$\begin{array}{ccc} \frac{d[D]}{dt} & = & \left(\lambda_{DC}\left[C\right]+\lambda_{DH}\left[H\right]\right)\left[D_{N}\right]-\left(\delta_{DC}\left[C\right]+\delta_{D}\right)\left[D\right]. \end{array}$$ (6)

### Macrophages (*M*)

Macrophages have many phenotypes and can change them frequently. For simplicity, we avoid the break down of them into M1, M2, and other subsets, and we model all activated macrophages as a single variable denoted by *M*. Tumor associated macrophages (TAMs) are activated by IL-10 [93–95]. Moreover, IL-12 activates M1 Macrophages [68, 93, 96–98], while M2 macrophages are activated by helper T-cells secreted cytokines (IL-13 and IL-4) [93].

Denoting naive macrophages by *M*_*N*_, activated macrophages by *M*, and the production rate of naive macrophages by *A*_*M*_, we can write the following system of equations for the dynamics of naive and activated macrophages.

$$\frac{d[M_{N}]}{dt}=A_{M}-(\lambda_{MIL_{10}}\left[IL_{10}\right]+\lambda_{MIL_{12}}\left[IL_{12}\right]+\lambda_{MT_{h}}\left[T_{h}\right])\left[M_{N}\right]-\delta_{M_{N}}\left[M_{N}\right],$$ (7)

$$\frac{d[M]}{dt}=(\lambda_{MIL_{10}}\left[IL_{10}\right]+\lambda_{MIL_{12}}\left[IL_{12}\right]+\lambda_{MT_{h}}\left[T_{h}\right])\left[M_{N}\right]-\delta_{M}\left[M\right].$$ (8)

### Cancer cells (*C*)

Traditionally, it is assumed that the growth rate of cancer cells is related to both the existing population and the available resources or space. Therefore, we model the proliferation of cancer cells by the logistic term [*C*](1 − [*C*]/*C*_0_), where *C*_0_ is the maximum capacity. In addition, IL-6 promotes the proliferation of cancer cells [99–101]. Also, adipocytes, releasing metabolic substrates, promote proliferation of breast cancer cells [102, 103]. On the other hand, activated CD8+ T-cells kill cancer cells [66, 104, 105]. The dynamics of cancer cells is modeled by the following equation.

$$\begin{array}{c} \frac{d[C]}{dt}=(\lambda_{C}+\lambda_{CIL_{6}}\left[IL_{6}\right]+\lambda_{CA}\left[A\right])\left(1-\frac{\left[C\right]}{C_{0}}\right)\left[C\right]-\left(\delta_{CT_{c}}\left[T_{c}\right]+\delta_{C}\right)\left[C\right]. \end{array}$$ (9)

### Cancer associated adipocytes (*A*)

Adipocytes participate in a highly complex cycle orchestrated by cancer cells to promote tumor progression [106]. Therefore, after including them in Eq (9), for simplicity we consider an independent logistic model describing their dynamics.

$$\begin{array}{c} \frac{d[A]}{dt}=\lambda_{A}\left[A\right](1-\frac{\left[A\right]}{A_{0}})-\delta_{A}\left[A\right]. \end{array}$$ (10)

### Necrotic cells (*N*)

Cells in the tumor microenvironment can die and turn into necrotic cells due to depletion of resources. This process is called necrosis and is known as a feature of tumors possessing an aggressive phenotype [107]. Necrotic cell death can replace other types of death for some cell types [45, 91]. As mentioned, activated CD8+ T-cells kill cancer cells [66, 104, 105] and we assume that death of cancer cells is the primary source of necrosis. Since a fraction of cancer cells can go through first becoming necrotic cells, the production rate of necrotic cells is modeled by the fraction (*α*_*NC*_) of dying cancer cells.

$$\begin{array}{c} \frac{d[N]}{dt}=\alpha_{NC}(\delta_{CT_{c}}\left[T_{c}\right]+\delta_{C})\left[C\right]-\delta_{N}\left[N\right]. \end{array}$$ (11)

### Molecules

In this section, we describe ODEs that govern dynamics of molecules in our model.

#### HMGB1 (*H*)

High-mobility group box 1 (HMGB1) is known to be a prototypical damage-associated molecular pattern (DAMP) protein, which alarms the body about disturbances in homeostasis [108]. HMGB1 exert immune promoting activity by inducing angiogenesis, proliferation, and invasiveness of cancer cells [90]. HMGB1 is mainly produced by dendritic cells [90, 91, 109, 110], necrotic cells [66, 109, 111, 112], macrophages, [109, 113–115], natural killer (NK) cells which behave like cytotoxic T-cells [109, 116–118], and cancer cells [66, 90, 91].

Therefore, the dynamics of HMGB1 is modeled by the following equation.

$$\begin{array}{c} \frac{d[H]}{dt}=\lambda_{HD}\left[D\right]+\lambda_{HN}\left[N\right]+\lambda_{HM}\left[M\right]+\lambda_{HT_{c}}\left[T_{c}\right]+\lambda_{HC}\left[C\right]-\delta_{H}\left[H\right]. \end{array}$$ (12)

#### IL-12 (*IL*_12_)

IL-12, stimulates the differentiation of naive T-cells into helper T-cells. Macrophages and dendritic cells secrete IL-12 [66, 86, 97, 119]. Also, IL-12 is produced by Helper and cytotoxic T-cells [120]. We model the dynamics of IL-12 using the following equation.

$$\begin{array}{c} \frac{d[IL_{12}]}{dt}=\lambda_{IL_{12}M}\left[M\right]+\lambda_{IL_{12}D}\left[D\right]+\lambda_{IL_{12}T_{h}}\left[T_{h}\right]+\lambda_{IL_{12}T_{c}}\left[T_{c}\right]-\delta_{IL_{12}}\left[IL_{12}\right]. \end{array}$$ (13)

#### IL-10 (*IL*_10_)

IL-10 is secreted by macrophages [93, 121, 122], dendritic cells [86, 123–125], T-reg cells [83, 126], CD4+ helper T-Cells [120, 127, 128], CD8+ cytotoxic T-cells [120, 126, 128], and cancer cells [87, 129]. Therefore, the dynamics of IL-10 is modeled in the following way.

$$\begin{array}{c} \begin{array}{cc} \frac{d[IL_{10}]}{dt} & =\lambda_{IL_{10}M}\left[M\right]+\lambda_{IL_{10}D}\left[D\right]+\lambda_{IL_{10}T_{r}}\left[T_{r}\right]+\lambda_{IL_{10}T_{h}}\left[T_{h}\right]+\lambda_{IL_{10}T_{c}}\left[T_{c}\right]\\ & +\lambda_{IL_{10}C}\left[C\right]-\delta_{IL_{10}}\left[IL_{10}\right]. \end{array} \end{array}$$ (14)

#### IL-6 (*IL*_6_)

The key cytokine that promotes the growth of cancer cells is IL-6 and is produced by cancer associated adipocytes [97, 99, 100, 130], macrophages [66, 93, 97, 98, 131, 132], and dendritic cells [66, 86, 120].

$$\begin{array}{c} \frac{d[IL_{6}]}{dt}=\lambda_{IL_{6}A}\left[A\right]+\lambda_{IL_{6}M}\left[M\right]+\lambda_{IL_{6}D}\left[D\right]-\delta_{IL_{6}}\left[IL_{6}\right]. \end{array}$$ (15)

### Mouse data analysis

**Fig 2:**
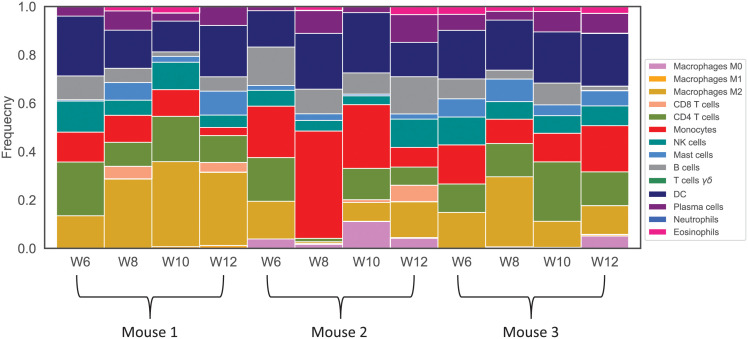
Immune cell frequencies for each mouse at different time points. Results acquired from deconvolution of gene expression data at each time point using CIBERSORTx B-mode.

For this study, we use the PyMT mice RNA-sequencing data available in the Gene Expression Omnibus (GEO) database as GSE76772 [71]. The PyMT gene expressions were acquired from 3 PyMT mice at four tumor progression stages: hyperplasia at week 6, adenoma/MIN at week 8, early carcinoma at week 10, and late carcinoma at week 12. The original study was designed to recognize gene expression similarities at different cancer stages. They used a directional RNA sequencing method to acquire the raw gene expression data. Later, they used statistical methods to remove transcriptionally inactive genes and get high confident normalized gene counts. We apply CIBERSORTx B-mode with the LM22 signature matrix [133] on the mentioned time-course gene expression data to estimate the relative abundance of each immune cell type in the tumor. Finally, we use expression values of genes encoding cytokines in the model and combined some immune cells to estimate the values of the model’s variables. Fig 2 shows the most variant immune cell frequencies for three mice at different time points. Since the deconvolution method only provides us the percentage of each immune cell type in primary tumors, we use the tumor size for each mouse to estimate the number of immune cells, cancer cells, and necrotic cells in each sample. Also, based on our observations, the average ratio of cancer cells to immune cells and necrotic cells is approximately 0.955:0.04:0.005 in mouse model breast tumors. Also, the epithelial cells density has been reported as 45 cells/mm^3^ in breast cancer [134]. Thus by choosing the scaling factor *α* = 45, the average density of cancer cells across all samples at each time is close to that value. So, we first calculate the total number of cells (TNC) for each mouse at a time point using

$${\text{TNC}}_{i}=\alpha \frac{\text{tumor}\;\text{size}(t_{i})}{\frac{1}{4}\sum_{i=1}^{4}\text{tumor}\;\text{size}(t_{i})}.$$

where *t*_*i*_ ∈ {6 weeks, 8 weeks, 10 weeks, 12 weeks} for *i* = 1, ⋯, 4. Using the TNC, we calculate the total number of cancer cells (TNCC), the total number of immune cells (TNIC), and the total number of necrotic cells (TNNC), using the ratio 0.955:0.04:0.005 and the following formulas

$${\text{TNIC}}_{i}=0.04\alpha \frac{\sum_{i=1}^{4}\text{Immune}\;\text{Cells}\;\text{Ratio}(t_{i})}{\frac{1}{4}\sum_{i=1}^{4}\text{Immune}\;\text{Cells}\;\text{Ratio}\left(t_{i}\right)}$$

$${\text{TNCC}}_{i}=\frac{191}{192}\left({\text{TNC}}_{i}-{\text{TNIC}}_{i}\right)\;\;\text{and}\;{\text{TNNC}}_{i}=\frac{{\text{TNCC}}_{i}}{191}.$$

See Table 2 for the values. The fraction $\frac{191}{192}$ is just the simplified ratio of cancer cells to necrotic cells 0.955 : 0.005.

**Table 2:** Cells and molecules values for each mouse at different time points.

**Mouse 1**	**Time** (days)	**T_N_**	**T_h_**	**T_C_**	**T_r_**	**D_N_**	**D**	**M_N_**	**M**
	0	18.91552	0.180307	0.001385	0.120725	13.04028	0.498882	6.767523	7.386616
	14	6.771898	0.673464	4.279462	0.001385	8.607666	0.001385	6.115784	15.72747
	28	16.02047	0.001385	0.164525	0.269144	6.955235	0.001385	6.053538	19.64027
	42	8.807364	0.073352	2.217549	0.001385	11.31217	0.313571	1.842909	17.24431
	**Time** (days)	**C**	**N**	**A**	**H**	**IL12**	**IL10**	**IL6**	
	0	6.56815	0.034388	93.82247	1000	28	417	2490	
	14	32.33359	0.169286	84.35702	940	24	351	1766	
	28	57.61312	0.301639	98.2119	1103	0	404	1599	
	42	83.48514	0.437095	83.62523	1050	2	455	1880	
**Mouse 2**	**Time** (days)	**T_N_**	**T_h_**	**T_C_**	**T_r_**	**D_N_**	**D**	**M_N_**	**M**
	0	14.18682	0.001459	0.001459	0.001459	8.67344	0.001459	14.50968	9.00316
	14	2.54051	0.787626	0.001459	0.001459	13.30604	0.001459	26.56551	0.632713
	28	7.022259	0.444987	2.773333	0.001459	14.39057	0.001459	21.57876	4.581648
	42	11.05377	0.001459	3.951772	0.001459	8.180317	0.001459	7.14771	8.669383
	**Time** (days)	**C**	**N**	**A**	**H**	**IL12**	**IL10**	**IL6**	
	0	6.590515	0.034505	92.75787	1182	0	450	1602	
	14	32.26456	0.168924	87.67355	932	16	723	1068	
	28	57.54278	0.301271	101.5889	945	0	429	1646	
	42	83.60215	0.437708	78.01466	807	0	319	1490	
**Mouse 3**	**Time** (days)	**T_N_**	**T_h_**	**T_C_**	**T_r_**	**D_N_**	**D**	**M_N_**	**M**
	0	10.84328	0.014559	2.398143	0.14663	11.10196	0.466044	9.302918	8.559201
	14	11.18575	0.748596	0.235111	0.001456	11.90139	0.001456	6.080938	16.72823
	28	17.73009	0.001456	0.608837	0.001456	12.19168	0.001456	6.812254	6.448463
	42	10.89853	1.288033	0.552229	0.001456	12.57453	0.001456	13.96522	7.217389
	**Time** (days)	**C**	**N**	**A**	**H**	**IL12**	**IL10**	**IL6**	
	0	6.73803	0.035278	85.66546	1521	19	511	3327	
	14	32.13758	0.16826	93.76585	1549	3	566	3481	
	28	57.83445	0.302798	87.59139	957	0	349	1716	
	42	83.28994	0.436073	92.99769	779	4	278	1490	

Time unit is represented in days. Although sampling starts at 6 weeks, we take that to be the origin of time, i.e. *t* = 0.

### Parameter estimation

For better stability, we perform the optimization process on the non-dimensionalized system, see the Non-dimensionalization appendix. In the following, for easier identification, we present matrix and vector quantities using boldface upper and lower case symbols, respectively.

#### Least-squares optimization

The set of parameters, i.e., the coefficients, proliferation, and death rates, when no specified bounds are desired, can be evaluated by re-arranging the 15 ODEs expressed in Eqs (1)–(15), and solving a linear least-squares problem via the following formula

$$\begin{array}{c} \theta ={\left(A^{T}A\right)}^{-1}A^{T}b, \end{array}$$ (16)

where **A** is the augmented matrix of mice’ data obtained by re-arranging, whose element *ij* is the *i*th observation of the *j*th variable, i.e., the value of each variable at specific time reported in Table 2. The rates are evaluated using a central finite difference method and are collected in vector **b**. The solution vector, ***θ***, provides the approximation for the 57 model parameters. The values of the carrying capacities *C*_0_ and *A*_0_ are determined based on the data, thus are considered known quantities, which keeps the system linear. In this study the parameters of the system are all non-negative; however, the expression in Eq (16) does not enforce any bounds on the solution that may result in negative values for the parameters. To enforce non-negativity, the following linear least-squares optimization problem is solved

$$\begin{array}{cc} \text{Find}: & \theta_{1},\cdots ,\theta_{57}\\ \underset{\theta }{\text{Minimize}:} & \frac{1}{2}{\left\|A\theta -b\right\|}_{2}^{2}\\ \text{Subject}\;\text{to}: & \theta_{min}\leq \theta_{e},\;e=1,\cdots ,57 \end{array}$$ (17)

that finds the solutions in the feasible region. The parameters’ bounds are the only constraint imposed. In this study, we solve the optimization problem above by setting *θ*_*min*_ = 10^−5^. In Eq (17), the solution is found for the minimum residual value in an iterative process where no inversion, such as the one in Eq (16), is needed. This approach of finding a solution using optimization (i.e., the iterative process) has been employed successfully in prior studies [135, 136]. See the Parameter values appendix where the optimized set of non-negative parameters are reported in Table 3.

**Table 3:** Non-dimensional parameter values.

Parameter	Value	Parameter	Value	Parameter	Value
${\bar{\lambda }}_{T_{h}H}$	1.0767 ⋅ 10^−5^	${\bar{\lambda }}_{T_{h}D}$	2.0501 ⋅ 10^−4^	${\bar{\lambda }}_{T_{h}IL_{12}}$	1.0751 ⋅ 10^−5^
${\bar{\lambda }}_{T_{c}D}$	0.0208	${\bar{\lambda }}_{T_{c}IL_{12}}$	1.0123 ⋅ 10^−5^	${\bar{\lambda }}_{T_{r}D}$	9.4550 ⋅ 10^−5^
${\bar{\lambda }}_{DC}$	0.0014	${\bar{\lambda }}_{DH}$	1.0484 ⋅ 10^−5^	${\bar{\lambda }}_{MIL_{10}}$	1.3208 ⋅ 10^−5^
${\bar{\lambda }}_{MIL_{12}}$	1.3208 ⋅ 10^−5^	${\bar{\lambda }}_{MT_{h}}$	1.0875 ⋅ 10^−5^	${\bar{\lambda }}_{C}$	0.0063
${\bar{\lambda }}_{CIL_{6}}$	2.1514 ⋅ 10^−4^	${\bar{\lambda }}_{CA}$	6.0466 ⋅ 10^−4^	${\bar{\lambda }}_{A}$	0.0024
${\bar{\lambda }}_{HD}$	0.0753	${\bar{\lambda }}_{HN}$	4.0155 ⋅ 10^−5^	${\bar{\lambda }}_{HM}$	5.5234 ⋅ 10^−4^
${\bar{\lambda }}_{HT_{c}}$	1.8188 ⋅ 10^−4^	${\bar{\lambda }}_{HC}$	4.0155 ⋅ 10^−5^	${\bar{\lambda }}_{IL_{12}M}$	1.0604 ⋅ 10^−5^
${\bar{\lambda }}_{IL_{12}D}$	5.8560 ⋅ 10^−5^	${\bar{\lambda }}_{IL_{12}T_{h}}$	0.0084	${\bar{\lambda }}_{IL_{12}T_{c}}$	7.1527 ⋅ 10^−4^
${\bar{\lambda }}_{IL_{10}M}$	1.2340 ⋅ 10^−5^	${\bar{\lambda }}_{IL_{10}D}$	2.6518 ⋅ 10^−4^	${\bar{\lambda }}_{IL_{10}T_{r}}$	8.4369 ⋅ 10^−4^
${\bar{\lambda }}_{IL_{10}T_{h}}$	0.0011	${\bar{\lambda }}_{IL_{10}T_{c}}$	1.0831 ⋅ 10^−5^	${\bar{\lambda }}_{IL_{10}C}$	1.0842 ⋅ 10^−5^
${\bar{\lambda }}_{IL_{6}A}$	4.1087 ⋅ 10^−4^	${\bar{\lambda }}_{IL_{6}M}$	1.4458 ⋅ 10^−4^	${\bar{\lambda }}_{IL_{6}D}$	0.0011
$\delta_{T_{h}}$	1.1053 ⋅ 10^−5^	$\delta_{T_{c}}$	1.0189 ⋅ 10^−5^	$\delta_{T_{r}}$	4.9935 ⋅ 10^−5^
$\delta_{T_{N}}$	1.0473 ⋅ 10^−5^	$\delta_{D_{N}}$	1.1051 ⋅ 10^−5^	*δ*_*D*_	0.3212
$\delta_{M_{N}}$	2.8489 ⋅ 10^−4^	*δ*_*M*_	1.0238 ⋅ 10^−5^	*δ*_*C*_	0.0032
*δ*_*A*_	0.0024	*δ*_*N*_	0.0013	*δ*_*H*_	0.0012
$\delta_{IL_{12}}$	0.0107	$\delta_{IL_{10}}$	0.0012	$\delta_{IL_{6}}$	0.0013
$\delta_{T_{h}T_{r}}$	0.0414	$\delta_{T_{h}IL_{10}}$	1.0753 ⋅ 10^−5^	$\delta_{T_{c}T_{r}}$	0.0162
$\delta_{T_{c}IL_{10}}$	1.0333 ⋅ 10^−5^	*δ*_*DC*_	1.7530 ⋅ 10^−4^	$\delta_{CT_{c}}$	9.9663 ⋅ 10^−5^
${\bar{A}}_{T_{N}}$	1.4442 ⋅ 10^−4^	${\bar{A}}_{D_{N}}$	5.8773 ⋅ 10^−4^	${\bar{A}}_{M}$	4.1504 ⋅ 10^−5^

### Sensitivity analysis

Sensitivity analysis is generally used to assess the sensitivity of the model’s output to system parameters [137]. To identify the most crucial parameters affecting the dynamics of cancer and the total number of cells, we perform sensitivity analysis on these quantities.

We use $\bar{x}$ to show non-dimensional variables. For a generic ODE system of the form

$$\begin{array}{c} \frac{d\bar{x}}{dt}=f\left(\bar{x},t,\theta \right) \end{array}$$ (18)

where $\bar{x}=\langle \bar{x_{1}},\cdots ,\bar{x_{15}}\rangle$ and ***θ*** = 〈*θ*_1_, ⋯, *θ*_57_〉 are vectors of state variables and parameters of our model, respectively, the first order local sensitivity of a variable ${\bar{x}}_{j}$ with respect to a parameter *θ*_*i*_ is evaluated by

$$\begin{array}{c} s_{i}=\frac{d{\bar{x}}_{j}}{d\theta_{i}},\;i=1,\cdots ,57 \end{array}$$ (19)

To obtain the sensitivity vector, **s** = 〈*s*_1_, ⋯, *s*_*M*_〉, we use a direct differential method. That is, we differentiate Eq (18) with respect to *θ*_*i*_ to get

$$\begin{array}{c} \frac{d}{dt}\frac{\partial \bar{x}}{\partial \theta_{i}}=\frac{\partial f}{\partial \bar{x}}\frac{\partial \bar{x}}{\partial \theta_{i}}+\frac{\partial f}{\partial \theta_{i}} \end{array}$$ (20)

and then we use a forward Euler discretization in time for Eq (20) to find $s_{i}=\partial \bar{x}/\partial \theta_{i}$. The sensitivity of each parameter in the neighborhood of a chosen parameter set Ω(*θ*) is then defined as

$$\begin{array}{c} {\hat{s}}_{i}=\int_{\Omega (\theta )}s_{i}d\theta . \end{array}$$ (21)

This neighborhood is created by perturbing our original parameter set by 10% and the integration is carried out numerically with sparse grid points [138, 139].

In this study, because some of our state variables do not reach steady-state within an experimentally reasonable time interval, we use a direct differential method rather than a steady-state method. As mentioned before, the latest data point was extracted at 12 weeks. However, our observations show that we need to continue the simulation for much longer than experimentally feasible so that all variables reach the steady-state. For this reason, we use a direct differential approach up to 18 weeks to obtain the sensitivities.

## Results

### Dynamics

**Fig 3:**
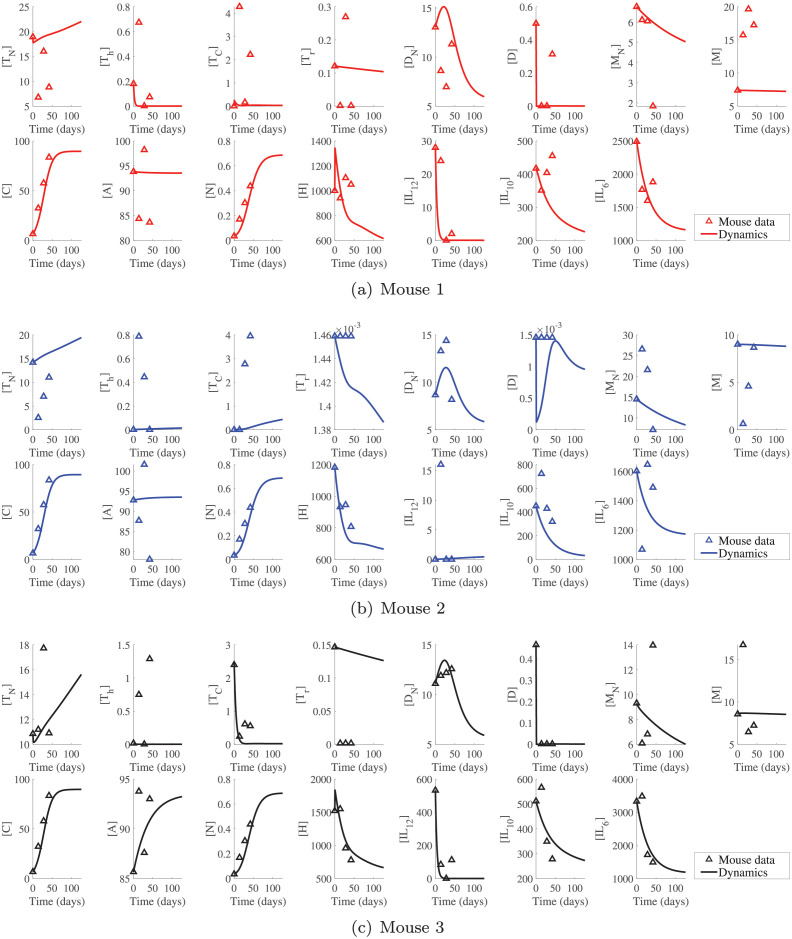
Comparison of the dynamics and mouse data. By solving the system of ODEs, the mouse data are compared to the values estimated using the optimal parameters obtained from the least-squares optimization. Time zero corresponds to 6 weeks which is the beginning of the mice data sampling.

We use the optimized parameters from the least-squares optimization and substitute them in the system of ODEs to obtain the dynamics of the system variables. As mentioned before, mice data was collected at weeks 6, 8, 10, and 12 corresponding to 42, 56, 70, and 84 days. However, we shifted our time interval so that 6 weeks becomes the time origin (*t* = 0) and 12 weeks maps to 42 days. We also continue our ODE solutions further than 42 days (up to 126 days) to match our sensitivity analysis. Fig 3 shows a comparison between the solution of ODEs and mouse data for the time period of 126 days. For a clear comparison of the results, the solution of ODEs and mice data are shown in one plot.

Despite the fact that the dynamics of cancer and necrotic cells fit the data well, a few predictions are less in accordance with the data points due to considerable disparities in the available data for some state variables. This can be remedied by more time course data decreasing the noise. However, it is considered a limitation of our study at this point.

Based on ODE simulations, naive T-cells for mice 1 and 3 show a quick decrease and then increase, while mouse 2 is strictly increasing. Helper T-cells for mice 1 and 3 quickly decrease to a very small steady-state, while for mouse 2, it gently increases. Cytotoxic cells behave the same way as the helper T-cells, and regulatory T-cells are generally decreasing with a negligible change in Mouse 2 compared to the other mice. Generally, for T-cells, our simulations are not in a good agreement with the data. Mouse 3 shows the best agreement in naive and cytotoxic T-cells, mouse 2 in regulatory T-cells and mouse 1 in helper T-cells.

The ODE results show that naive dendritic cells increase and then decrease in all 3 mice. Activated dendritic cells sharply decrease to a small steady-state in mice 1 and 3. In mouse 2, we can see a fluctuation in dendritic cells, but these changes are very small and negligible and eventually settles at a small value like the other two mice. Compared to T-cells, dendritic cells show a much better agreement with data in all three mice. The number of naive macrophages decrease in all three mice. Activated macrophages also decrease with similar rates in all three mice. Again, the simulation results are not in good agreement with the data. Mouse 1 shows the best agreement in naive macrophages and, mouse 3 in activated macrophages.

The predicted dynamics of cancer cells in all three mice follow the data very closely; dynamics in all three mice are similar, and they reach a steady-state value within 75 days. Given the closeness of reported data points in three mice, this similarity in their behavior is expected. Adipocytes’ dynamics also reach the steady-state values in all three mice. They do so by slowly decreasing in mouse 1 and slowly increasing in mouse 2. Mouse 3 undergoes a sharper increase before it converges to its steady-state value. Interestingly, in all three mice, the number of adipocytes converges to the same value. In mouse 3, we see a fair agreement between the dynamic of adipocytes and the data unlike the other two mice. Necrotic cells, like cancer cells, show a logistic growth and a perfect match with the data. We see almost the same behavior in all 3 mice reaching the same steady-state value. This is expected since the source of necrosis in the model is the death of cancer cells.

HMGB1 dynamics show a good agreement with the data, in all three cases. They start with a sharp increase from the initial value followed by a fast decrease. IL12 dynamics in mice 1 and 3 quickly decrease to a steady-state, but mouse 2 shows a mild, strictly increasing behavior. Mouse 1 and 3 also follow the data closely unlike mouse 2. IL10 decreases in all cases with a rather steeper slope in mouse 2. As a matter of fact, mouse 2 is the only mouse for which IL10 reaches its steady-state value within 125 days. Finally, IL6 decreases to a steady-state within the simulation time for all three mice, with mouse 3 showing the steepest descend followed by mouse 1. In general, we see a fair correspondence between dynamics and data in all 3 mice for both IL10 and IL 6. However, this correspondence is better in mice 1 and 3 than mouse 2.

Now we comment on the interactions, which we believe are responsible for the differences in the dynamics. For activated T-cell subtypes, we can see from equations Eqs (1) and (2) that dendritic cells, IL12 and HMGB1 are involved in their promotion, and regulatory T-cells and IL10 are involved in their inhibition process.

Helper T-cells are produced by activation of naive T-cells and inhibited by T-reg cell. For helper T-cells, we can see that mouse 2 has significantly fewer T-reg cells in the long run, and its IL12 levels are increasing, unlike the other two mice. These two behaviors contribute to a rise in the number of helper T-cells in this mouse. The same goes for cytotoxic cells. T-reg cell levels are only dependent on dendritic cells. Dendritic cells in Mice 1 and 3 start at high values contributing to some activation of T-reg cells and then a quick depletion. This explains the decreasing of T-reg cells in these mice. As for mouse 2, the number of dendritic cells starts low and stays low, resulting in the low numbers of T-reg cells and the sharp decrease in this case. Finally, Eq (5) shows that in the model, naive T-cells are produced via a constant rate, while activation of other T-cell subtypes contributes to their depletion. All three mice show a growing trend for naive T-cells. Given that the other subtypes are generally low or depleting (mice 1 and 3) or have a very gentle growth (mouse 2), the natural production of naive T-cells dominates the process.

Dendritic cells are activated and inhibited by cancer cells, see Eq (6). Therefore, its activation by HMGB1 can be the key to the observed behaviors. The sudden decrease in HMGB1 in all mice can be the reason that the decaying effects in Eq (6) have taken over so quickly. For the naive dendritic cells, the sudden surge results from the sudden drop in activated dendritic cells. However, natural decay takes over later.

By looking at Eq (7), we can see that macrophages get activated by IL10, IL12, and helper T-cells, and the only cause of death considered for them in this model is their natural decay. All the activators decrease in mice 1 and 3. In mouse 2, we have a slight increase in helper T-cells and IL12, and maybe that is why mouse 2 has the highest level of activated macrophages. However, at the end, these effects are not enough to win over the natural decay, and hence we see an overall decrease in all three cases. For naive macrophages, we can see that the death rate in Table 3, is an order of magnitude larger than the natural production rate. As a result, there is a decrease in naive macrophages in all three mice.

In the model, either cancer cells production happens independently or is promoted by adipocytes and IL6. They can also die naturally or by cytotoxic cells. As mentioned before, adipocytes in all three mice converge to the same value. Also, IL6 behavior is similar across all cases, while cytotoxic cells follow a different pattern in mouse 2. In fact, the increase in *Tc* observed in mouse 2 agrees with CD8 frequency shown in Fig 2. Adipocytes and IL6 play crucial roles in the cancer dynamics given their roles and the similarity between cancer dynamics and their dynamics. Adipocytes are modeled independently. They follow a logistic population model, and depending on their estimated growth and decay rate; they increase or decrease and then saturate. Also, since necrotic cells are produced as a result of cancer cells’ death, their dynamics are self-explanatory.

HMGB1 is produced proportional to the number of dendritic cells, necrotic cells, macrophages, cytotoxic cells, and cancer cells and decays naturally. Among the mentioned cell types, cancer and necrotic cells are the only ones whose numbers increase in time, and the rest of the cell types decrease. But, the parameter estimation shows that cancer cells and necrotic cells have the smallest production rates (two orders of magnitude smaller than the natural decay rate). Therefore, HMGB1 won’t be affected by them and decreases in time.

IL12 is produced proportional to the number of dendritic cells, macrophages, cytotoxic and helper T-cells and decays naturally. Higher levels of macrophages and increasing levels of helper and cytotoxic T-cells in mouse 2 is the reason for its mild increase, unlike mice 1 and 3.

IL10 is produced proportional to the number of dendritic cells, macrophages, cytotoxic, helper, and regulatory T-cells plus cancer cells and decays naturally. Given its large production rate by helper T-cells and its even larger decay rate, this cytokine is more significantly affected by these two parameters. Therefore, it decays quickly in all mice. However, we can see its steady-state value in mice 2 within the simulation time interval. This is mostly due to the increasing helper T-cells, which dampens the decreasing effect of its natural decay.

Finally, IL6 is produced by adipocytes, macrophages, and dendritic cells and is removed naturally. The dynamics of IL-6 is heavily depend on its production rate by dendritic cells, because its production by dendritic cells and its natural decay rate are orders of magnitude larger than its production rates by adipocytes and macrophages. Hence, we see a strict decreasing trend in all three mice.

### Sensitivity analysis results

**Fig 4:**
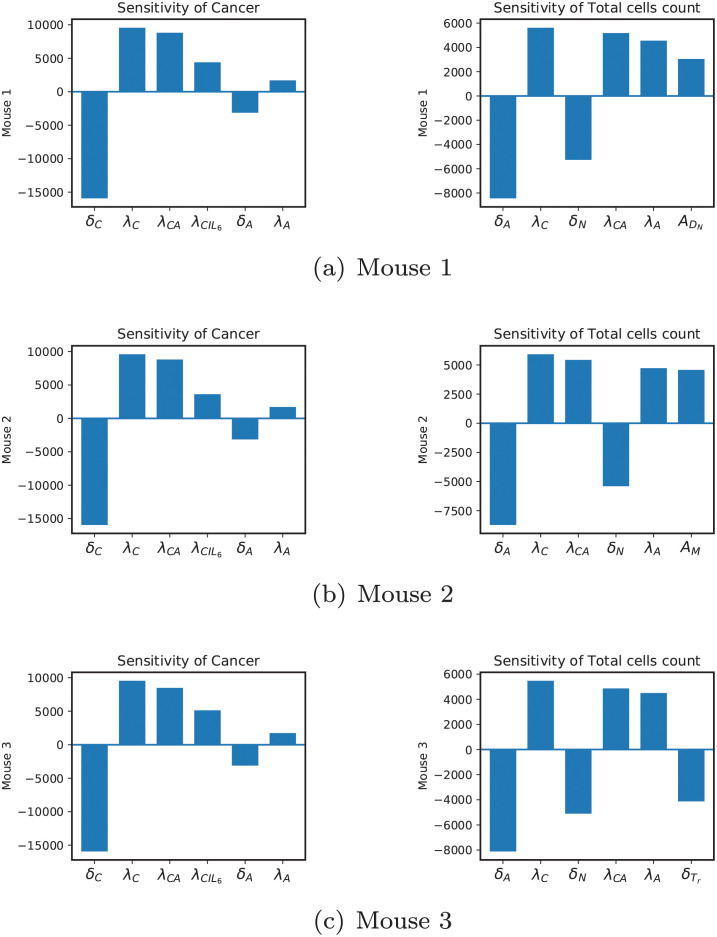
Sensitivity results. Sensitivity levels of the top 6 most sensitive parameters for cancer and total number of cells.

**Fig 5:**
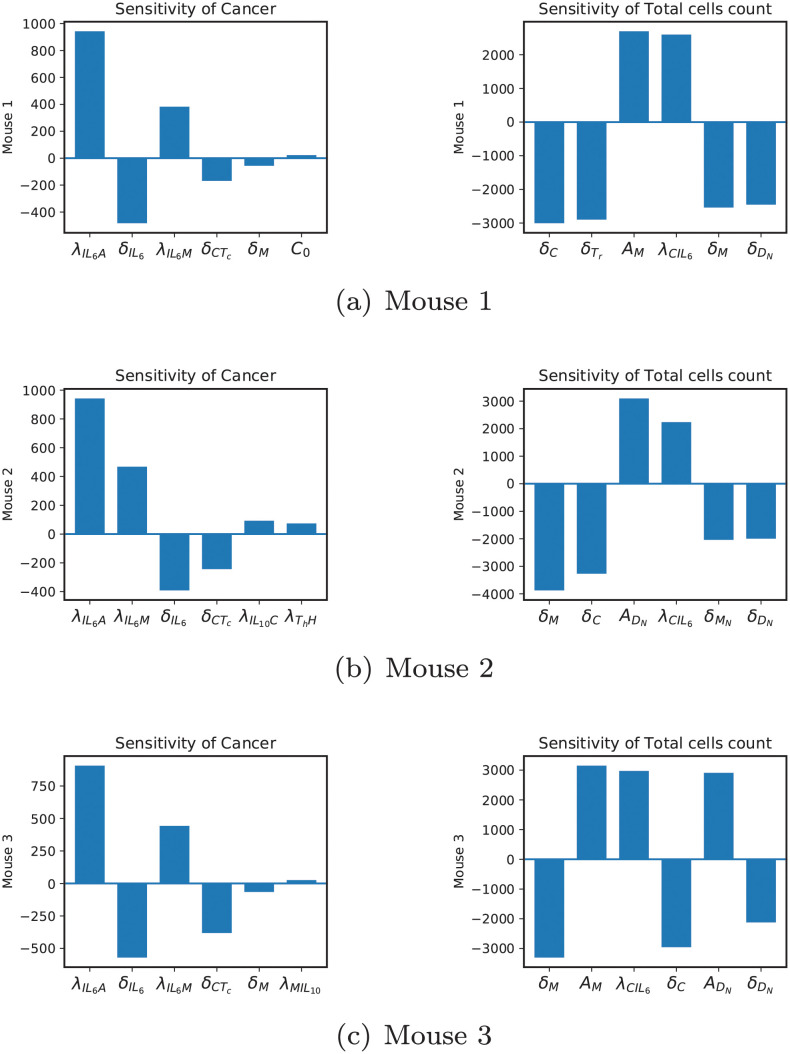
Sensitivity results. Other sensitive parameters for cancer and total number of cells.

Figs 4 and 5 show the results of the sensitivity analysis. A positive value for a parameter means its increase will directly affect the population of cancer or the total number of cells depending on the plot title, and a negative value means the opposite. Fig 4 shows the same sensitive parameters for cancer in all three mice. However, there are some differences when it comes to sensitivity for the total number of cells.

In all three mice, the natural decay rate of cancer cells is the most sensitive parameter. It is important to point out that calling it the natural decay rate of cancer is an abuse of terminology and is merely for convenience. In fact, in addition to natural death, *δ*_*C*_ includes the rate of cancer death caused by anything other than cytotoxic cells (which have been directly included in the model). This description engulfs a large set of biological reasons affecting the cancer population and is suitably recognized as the most sensitive parameter. Similarly, for λ_*C*_ being cancer proliferation rate promoted by anything other than adipocytes and IL6 (which have been directly included in the model). The next sensitive parameters for cancer are λ_*CA*_, and λ_*CIL*6_. As mentioned in the previous section, adipocytes and IL6 play a big role in cancer dynamics. Adding to that, we can see *δ*_*A*_ and λ_*A*_ are also among the most sensitive parameters and $\lambda_{IL_{6}A},\delta_{IL_{6}}$, and $\lambda_{IL_{6}M}$ are at top of the rest of sensitive parameters, see Fig 5. These imply that controlling adipocytes and IL6 (in that order) might be a gateway to controlling cancer proliferation in these mice. The removal rate of cancer cells by cytotoxic cells is the tenth sensitive parameter. Even though this rate is directly involved in the cancer ODE, it is not as sensitive as adipocyte and IL6 related parameters mentioned before. This might be due to the very low expression of cytotoxic cells in the mouse model. Next, for mice 1 and 3, we have *δ*_*M*_, and for mouse 2, we have $\lambda_{IL_{10}C}$ as sensitive parameters. Macrophages affect the cancer population indirectly by producing cytokines like IL6, IL10, and IL2. IL6 directly affects cancer dynamics, while IL10 and IL12 do it by affecting cytotoxic cells. For Mouse 2, the parameter $\lambda_{IL_{10}C}$ promotes the production of IL10, which can affect cancer through cytotoxic cells. The last set of parameters are *C*_0_ for mouse 1, $\lambda_{T_{h}H}$ for mouse 2 and $\lambda_{MIL_{10}}$ for mouse 3. Cancer cells’ carrying capacity dictates how far they can go before depleting their resources and are explicitly involved in cancer ODE. Production of HMGB1 by *T*_*h*_ can lead to cancer through several interactions, such as promoting the production of dendritic cells, which leads to more production of all cytokines, or through helper T-cells which are similarly cytokine producers. The fastest route that connects the production of IL10 by macrophages is the route that leads to cytotoxic cells. The more IL10, the more removal of cytotoxic cells and hence less death of cancer cells by cytotoxic cells.

Before discussing the sensitive parameters for total cells, it is important to note that *T*_*N*_ is excluded from the total cell count, because they are not primarily present in the tumor microenvironment and are frequently detected in the circulation and lymphatic system [75]. Other T-cells get activated and infiltrate the tumor and can be found in copious amounts in tumors. Dendritic cells get activated inside of the tumor, and cancer cells, necrotic cells, and adipocytes are the other components of the breast tumor [140]. Finally, since most of the naive macrophages polarize into different phenotypes inside of the tumor, we include a 20% factor for *M*_*N*_ [141]. Therefore, we have:

$$\begin{array}{c} \text{Total}\;\text{Cells}=T_{h}+T_{c}+T_{r}+D_{N}+D+0.2M_{N}+M+C+N+A \end{array}$$ (22)

The total number of cells is an important measurement directly related to the size of the tumor. Sensitivity results show that parameters *δ*_*A*_, λ_*C*_, *δ*_*N*_, λ_*CA*_, and λ_*A*_ are included as top 5 sensitive parameters in all 3 mice in Fig 4. Four out of five of these parameters govern the population of adipocytes and cancer cells. These results are reasonable given that they have the largest populations among all other cells. However, the necrosis related parameter is not as straightforward, since we do not have a large number of necrotic cells in these tumors. If we track the influence of necrotic cells in the model, we see that they only contribute to the production of HMGB1. This cytokine is involved in the dynamics of helper T-cells and naive and activated dendritic cells. The sixth sensitive parameters are $A_{D_{N}}$ for mouse 1, *A*_*M*_ for mouse 2 and $\delta_{T_{r}}$ for mouse 3. This difference is interesting since mouse 1 has the highest amount of naive dendritic cells, mouse 2 has the largest number of macrophages, and mouse 3 has the most regulatory T-cells. The rest of the sensitive parameters in Fig 5 are independent death rates or production rates of cells that are present in the tumor’s microenvironment and can be justified similar to above. However, we notice the presence of $\lambda_{CIL_{6}}$ again. As λ_*CA*_ and $\lambda_{CIL_{6}}$ play a crucial role in cancer proliferation, they are also important in controlling the size of the tumor.

### Varying dynamics and bifurcation

**Fig 6:**
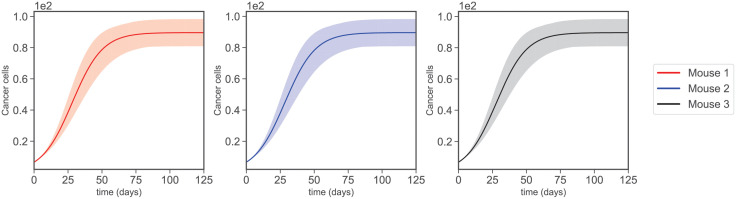
Varying dynamics. The transparent regions are acquired by 20% perturbation of all the sensitive parameters in Figs 4 and 5.

This section further explores the effect of parameters on cancer dynamics. First, we perturb all sensitive parameters of cancer by 20% to see the collective effect of changing these parameters. Fig 6 shows that all three mice almost identically respond to these perturbations. This indicates good stability of the parameter estimations, especially since this has been acquired by the perturbation of parameters that have the biggest impact on the model dynamics.

**Fig 7:**
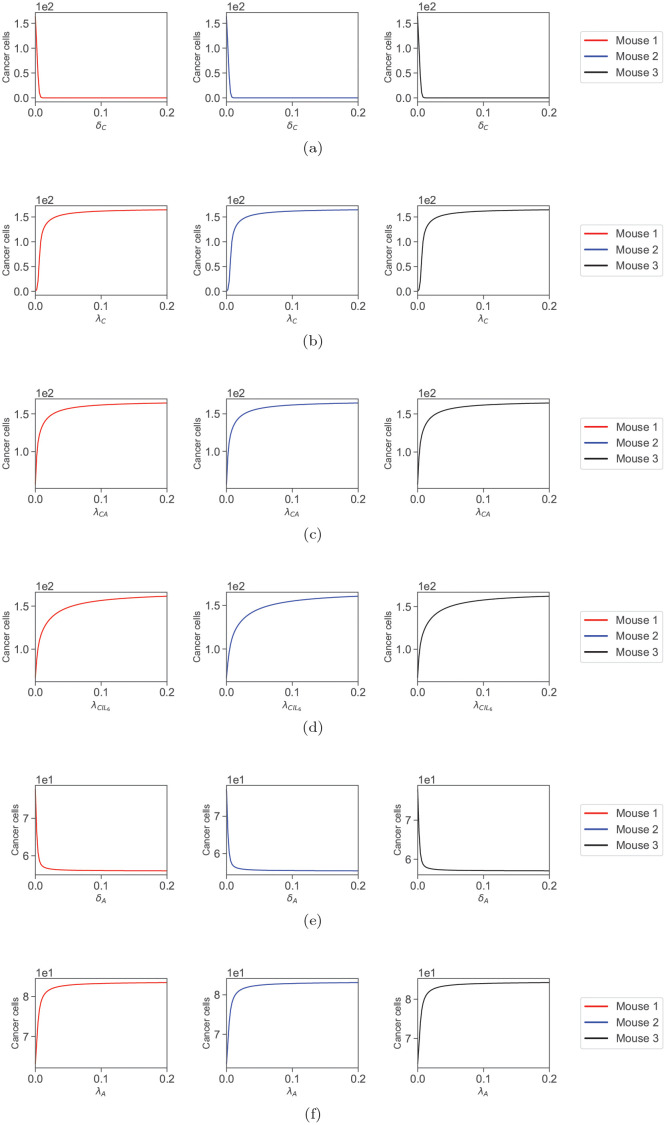
Bifurcation plots. Bifurcation of cancer values after 12 weeks with respect to the most sensitive parameters in Fig 4, namely (a)*δ*_*C*_ (b) λ_*C*_ (c) λ_*CA*_ (d) $\lambda_{CIL_{6}}$ (e) *δ*_*A*_ and (f) λ_*A*_.

Finally, we explore the local effect of the top 6 sensitive parameters (from Fig 4) for cancer on its dynamic. We do this by calculating the value of cancer at *t* = 42 days with respect to each sensitive parameter separately with the rest of the parameter values being fixed. As a reminder, we take 6 weeks which is the beginning of the mouse sampling, to be *t* = 0 days, and that makes *t* = 42 days corresponding to 12 weeks. As mentioned before, some state variables reach the steady-state very late; therefore, we limited the bifurcation points to the level of cancer at the last sampling time (12 weeks). The benefit of these results is that we can investigate the independent effect of large changes in single parameter values. We choose the interval [0, 0.2] for all the target parameters. Among sensitive parameters, λ_*C*_ has the largest estimated value of 0.0063, and the choice of the interval [0, 0.2] covers values 30 times larger than this value. Note that there is no limitation in choosing the interval, and this choice has only been made for better scaling and visual purposes (see Fig 7). In other words, the plots in Fig 7 are a zoomed-in and cropped version of more extensive bifurcation diagrams, as there are branches for negative values and chaos regions for large values that are not biologically realistic parameters’ value regimes.

Again, all three mice show almost identical behaviors. By increasing death rate values such as *δ*_*C*_ and *δ*_*A*_, we can significantly reduce the value of cancer cells at the last sampling time. As mentioned before, *δ*_*C*_ has a rather obscure meaning as it can be the death of cancer cells promoted by any reasons not directly included in the model. However, we can specifically see that removing adipocytes by significantly increasing their death rate leads to a notable reduction in the population of cancer cells at the last sampling time, see Fig 7a and 7e.

On the other hand, increasing the production rates such as λ_*C*_, λ_*CA*_, $\lambda_{CIL_{6}}$, and λ_*A*_ increases the cancer population at the last sampling time. Among these, λ_*A*_ has the smallest effect, but the others can cause the cancer population to reach double its last value in Fig 3. Also, λ_*C*_ has the same obscurity as *δ*_*C*_, since it engulfs the production rate of cancer promoted by reasons other than what we have already included in the model. But λ_*CA*_, $\lambda_{CIL_{6}}$ and λ_*A*_, specify that controlling the processes for which cancer production is promoted by IL6 and adipocytes or even reducing the production of these two can lead to a better result.

## Discussion

In this study, we modeled the breast cancer progression in PyMT mice using a system of ODEs. Biologically, cancer is an intricate interaction network with many cells and molecules involved in its development. For the model, we identified key players and devised a simplified interaction network based on the available literature, see Fig 1. To further reduce the complexity of the model, we mostly used simple mass action kinetics and linear ODEs, except for cancer and adipocytes that follow a logistic growth model, see Eqs (1)–(15).

We acquired the gene expression data from the PyMT RNA-sequencing data available in the Gene Expression Omnibus (GEO) database as GSE76772 [71]. These data were collected from 3 PyMT mice after 6, 8, 10, and 12 weeks. We used CIBERSORTx B-mode to deconvolute the gene expression data and obtained each mouse’s time course data set. We used these data to estimate all the parameters in the ODE system.

Although the dynamics shown in Fig 3 are not an excellent fit for all the variables, ODE solutions closely follow a couple of important variables such as cancer and necrotic cells. The mismatch between the data and dynamics can be attributed to lacking sufficient biological information. Furthermore, we need to continue the simulations way past the feasible experimental time for all dynamics to reach the steady-state. However, we can see the steady-state values for cancer and a few more state variables by continuing the response evaluation up to 126 days (three times are longer than the last mouse data sampling).

The simulations indicate similar attributes for mice 1 and 3. Mouse 2 showed similar trends in most variables except for helper, cytotoxic, regulatory T-cells, activated dendritic cells, and IL12, see Fig 3. Maybe the most interesting observation was that cancer dynamics were almost identical in all three mice despite these differences. We argued that the similarity in adipocytes and IL6 dynamics in three mice dominates the discrepancies in other variables. This was simply a hunch based on the direct mathematical involvement of these two variables in the cancer ODE. We further confirmed this by looking at sensitivity levels of cancer to all the parameters of the model. The observed differences in variables of mouse 2 from mice 1 and 3 might be due to the differences in the percentages of macrophages’ subtypes (Fig 2), because a high level of M2 macrophages can suppress cytotoxic T cells and inhibit anti-tumor immunity [142, 143].

We carried out a sensitivity analysis based on a direct differential method, and the results showed us that the cancer population in all three mice is sensitive to *δ*_*C*_, λ_*C*_, λ_*CA*_, $\lambda_{CIL_{6}}$, *δ*_*A*_ and λ_*A*_ in that order, see Fig 4. Commenting on the biological significance of *δ*_*C*_ and λ_*C*_ is rather difficult, since they can include the death and production of cancer promoted by cells and chemicals not included in the model. However, 3 out of 6 of these parameters are related to adipocytes, and looking at the rest of the sensitive parameters in Fig 5, we can also observe the importance of IL6 in cancer dynamics. We even investigated these parameters locally for much larger values through the bifurcation plots and saw regions for which cancer can be controlled through each of the sensitive parameters in Fig 5.

The link between obesity and breast cancer has been observed by many researchers. In 2007, about 7% of all new cases of cancer in women were related to obesity [144]. Obesity results in an elevated amount of adipose tissue (fat), and a direct relationship between excess fat and increased mortality rate in many types of cancer, including breast cancer, has been confirmed [145]. Prevention and medication approaches have been utilized to stop or reverse dysfunctional adipose tissue. Approaches such as weight loss strategies or medications such as metformin, statins, nonsteroidal anti-inflammatory drugs, and docosahexaenoic acid have been widely studied [146]. In addition, there are studies that suggest that leptin (a hormone produced by adipocytes) is involved in increasing breast cancer risk in postmenopausal women, and targeting it might be a key to controlling cancer in such patients [147, 148]. All of these confirm the importance of adipocytes in breast cancer development. Moreover, there are many studies recognizing IL6 as a key cytokine in progressive breast cancer, confirming that high levels of IL6 are related to poor breast cancer prognoses and showcasing its therapeutic significance in treating cancer patients [18, 19, 70]. Finally, it has been discussed that increased inflammation and IL-6 secretion in adipocytes, plus a hypoxic tumor microenvironment, creates an ideal opportunity for adipocyte-derived IL6 to promote angiogenesis [149]. So not only do adipocytes and IL6 independently contribute to poor breast cancer prognoses, but their combined effect has been acknowledged as a promoter of angiogenesis.

The approach chosen in this paper is one amongst many. There are many ways to model the interaction network. Many cells and cytokines have not been included in this study which have the potential to be integrated in our future studies. For example, our model does not consider resources such as oxygen or metabolites, macrophage heterogeneity, and the formation of blood vessels (angiogenesis). Similarly, we do not incorporate cancer stem cells, which are a tumor-initiating, self-renewing population typically resistant to therapeutics [150, 151]. The role of fibroblasts which tend to support the cancer cell niche [152], can also be considered in future iterations of the model. Furthermore, given the patient-to-patient heterogeneity, a mathematical model including more cell types and interactions mechanisms would require extensive time-course data and underlying parameters that describe these interactions. As mentioned in our manuscript, the lack of sufficient time-course data is a significant limitation of our study. Therefore, there is much room for improvement in expanding the interaction system, the validation phase, or even the dimension of the problem. Nevertheless, the current approach will guide our future studies to build targeted treatment models that focus on suppressing adipocytes and IL6. There are already studies targeting specific proteins or signaling patterns by adipocytes to control cancer [153, 154]. Also, high levels of adipocytes lead to up-regulated IL6, which build resistance to anti-VEGF therapy in breast cancer [155]. These suggest attractive therapy models with resistance terms for our future work.

### Non-dimensionalization

We non-dimensionalize the system of ODEs by dividing each variable by its maximum value over all mice and time points for more stable numerical simulation, parameter estimation, and sensitivity analysis. As a result, the steady-state value of a non-dimensional variable $\left[\bar{X}\right]$, which is [*X*]/[*X*^∞^], equals 1. Accordingly, the following system is obtained:

$$\begin{array}{cc} \frac{d[\bar{T_{h}}]}{dt} & =({\bar{\lambda }}_{T_{h}H}\left[\bar{H}\right]+{\bar{\lambda }}_{T_{h}D}\left[\bar{D}\right]+{\bar{\lambda }}_{T_{h}IL_{12}}\left[\bar{IL_{12}}\right])\left[\bar{T_{N}}\right]\\ & -({\bar{\delta }}_{T_{h}T_{r}}\left[\bar{T_{r}}\right]+{\bar{\delta }}_{T_{h}IL_{10}}\left[\bar{IL_{10}}\right]+\delta_{T_{h}})\left[\bar{T_{h}}\right], \end{array}$$ (23)

$$\frac{d[\bar{T_{c}}]}{dt}=({\bar{\lambda }}_{T_{c}D}\left[\bar{D}\right]+{\bar{\lambda }}_{T_{c}IL_{12}}\left[\bar{IL_{12}}\right])\left[\bar{T_{N}}\right]-({\bar{\delta }}_{T_{c}T_{r}}\left[\bar{T_{r}}\right]+{\bar{\delta }}_{T_{c}IL_{10}}\left[\bar{IL_{10}}\right]+\delta_{T_{c}})\left[\bar{T_{c}}\right],$$ (24)

$$\frac{d[\bar{T_{r}}]}{dt}={\bar{\lambda }}_{T_{r}D}\left[\bar{D}\right]\left[\bar{T_{N}}\right]-\delta_{T_{r}}\left[\bar{T_{r}}\right],$$ (25)

$$\begin{array}{cc} \frac{d[\bar{T_{N}}]}{dt} & ={\bar{A}}_{T_{N}}-({\bar{\lambda }}_{T_{h}H}\left[\bar{H}\right]+{\bar{\lambda }}_{T_{h}D}\left[\bar{D}\right]+{\bar{\lambda }}_{T_{h}IL_{12}}\left[\bar{IL_{12}}\right])\left[\bar{T_{N}}\right]\\ & -({\bar{\lambda }}_{T_{c}D}\left[\bar{D}\right]+{\bar{\lambda }}_{T_{c}IL_{12}}\left[\bar{IL_{12}}\right])\left[\bar{T_{N}}\right]\\ & -({\bar{\lambda }}_{T_{r}D}\left[\bar{D}\right]+\delta_{T_{N}})\left[\bar{T_{N}}\right], \end{array}$$ (26)

$$\frac{d[\bar{D_{N}}]}{dt}={\bar{A}}_{D_{N}}-({\bar{\lambda }}_{DC}\left[\bar{C}\right]+{\bar{\lambda }}_{DH}\left[\bar{H}\right])\left[\bar{D_{N}}\right]-\delta_{D_{N}}\left[\bar{D_{N}}\right],$$ (27)

$$\frac{d[\bar{D}]}{dt}=({\bar{\lambda }}_{DC}\left[\bar{C}\right]+{\bar{\lambda }}_{DH}\left[\bar{H}\right])\left[\bar{D_{N}}\right]-({\bar{\delta }}_{DC}\left[\bar{C}\right]+\delta_{D})\left[\bar{D}\right],$$ (28)

$$\frac{d[\bar{M_{N}}]}{dt}={\bar{A}}_{M}-({\bar{\lambda }}_{MIL_{10}}\left[\bar{IL_{10}}\right]+{\bar{\lambda }}_{MIL_{12}}\left[\bar{IL_{12}}\right]+{\bar{\lambda }}_{MT_{h}}\left[\bar{T_{h}}\right]+\delta_{M_{N}})\left[\bar{M_{N}}\right],$$ (29)

$$\frac{d[\bar{M}]}{dt}=({\bar{\lambda }}_{MIL_{10}}\left[\bar{IL_{10}}\right]+{\bar{\lambda }}_{MIL_{12}}\left[\bar{IL_{12}}\right]+{\bar{\lambda }}_{MT_{h}}\left[\bar{T_{h}}\right])\left[\bar{M_{N}}\right]-\delta_{M}\left[\bar{M}\right],$$ (30)

$$\frac{d[\bar{C}]}{dt}=(\lambda_{C}+{\bar{\lambda }}_{CIL_{6}}\left[\bar{IL_{6}}\right]+{\bar{\lambda }}_{CA}\left[\bar{A}\right])(1-\frac{\left[\bar{C}\right]}{\bar{C_{0}}})\left[\bar{C}\right]-({\bar{\delta }}_{CT_{c}}\left[\bar{T_{c}}\right]+\delta_{C})\left[\bar{C}\right],$$ (31)

$$\frac{d[\bar{A}]}{dt}=\lambda_{A}\left[\bar{A}\right](1-\frac{\left[\bar{A}\right]}{\bar{A_{0}}})-\delta_{A}\left[\bar{A}\right],$$ (32)

$$\frac{d[\bar{N}]}{dt}={\bar{\alpha }}_{NC}({\bar{\delta }}_{CT_{c}}\left[\bar{T_{c}}\right]+\delta_{C})\left[\bar{C}\right]-\delta_{N}\left[\bar{N}\right],$$ (33)

$$\frac{d[\bar{H}]}{dt}={\bar{\lambda }}_{HD}\left[\bar{D}\right]+{\bar{\lambda }}_{HN}\left[\bar{N}\right]+{\bar{\lambda }}_{HM}\left[\bar{M}\right]+{\bar{\lambda }}_{HT_{c}}\left[\bar{T_{c}}\right]+{\bar{\lambda }}_{HC}\left[\bar{C}\right]-\delta_{H}\left[\bar{H}\right],$$ (34)

$$\frac{d[\bar{IL_{12}}]}{dt}={\bar{\lambda }}_{IL_{12}M}\left[\bar{M}\right]+{\bar{\lambda }}_{IL_{12}D}\left[\bar{D}\right]+{\bar{\lambda }}_{IL_{12}T_{h}}\left[\bar{T_{h}}\right]+{\bar{\lambda }}_{IL_{12}T_{c}}\left[\bar{T_{c}}\right]-\delta_{IL_{12}}\left[\bar{IL_{12}}\right],$$ (35)

$$\begin{array}{cc} \frac{d[\bar{IL_{10}}]}{dt} & ={\bar{\lambda }}_{IL_{10}M}\left[\bar{M}\right]+{\bar{\lambda }}_{IL_{10}D}\left[\bar{D}\right]+{\bar{\lambda }}_{IL_{10}T_{r}}\left[\bar{T_{r}}\right]+{\bar{\lambda }}_{IL_{10}T_{h}}\left[\bar{T_{h}}\right]+{\bar{\lambda }}_{IL_{10}T_{c}}\left[\bar{T_{c}}\right]\\ & +{\bar{\lambda }}_{IL_{10}C}\left[\bar{C}\right]-\delta_{IL_{10}}\left[\bar{IL_{10}}\right], \end{array}$$ (36)

$$\frac{d[\bar{IL_{6}}]}{dt}={\bar{\lambda }}_{IL_{6}A}\left[\bar{A}\right]+{\bar{\lambda }}_{IL_{6}M}\left[\bar{M}\right]+{\bar{\lambda }}_{IL_{6}D}\left[\bar{D}\right]-\delta_{IL_{6}}\left[\bar{IL_{6}}\right].$$ (37)

Since we are not non-dimensionalizing with respect to the time, the production rates, λ_*C*_ and λ_*A*_, and the decay rates, $\delta_{T_{h}}$, $\delta_{T_{c}}$, $\delta_{T_{r}}$, $\delta_{T_{N}}$, $\delta_{D_{N}}$, *δ*_*D*_, $\delta_{M_{N}}$, *δ*_*M*_, *δ*_*C*_, *δ*_*A*_, *δ*_*N*_, *δ*_*H*_, $\delta_{IL_{12}}$, $\delta_{IL_{10}}$, and $\delta_{IL_{6}}$, are left unchanged.

### Parameter values

The values of the model parameters obtained from the least-squares optimization discussed in the section of Parameter estimation are reported in Table 3. The given constant values in the optimization process are *C*_0_ = 2, *A*_0_ = 2, and *α*_*NC*_ = 1.5.
